# Revisiting LSDMCA: male lethality escape and genotype-phenotype correlations

**DOI:** 10.1038/s41431-026-02098-7

**Published:** 2026-04-21

**Authors:** Alfonso Manuel D’Alessio, Alessia Indrieri, Giuseppina Vitiello, Manuela Morleo, Susan Schelley, Gregory M. Enns, Chiara Passarelli, Roberta Tammaro, Valeria Tiranti, Camille Peron, Wallid Deb, Antonio Novelli, Bertrand Isidor, Achille Iolascon, Brunella Franco

**Affiliations:** 1 Telethon Institute of Genetics and Medicine (TIGEM), Pozzuoli, Italy; 2 Scuola Superiore Meridionale, Genomic and Experimental Medicine program, Naples, Italy; 3 Unit of Medical Genetics, Department of Molecular Medicine and Medical Biotechnologies, (Dai Med Lab), Federico II University, Naples, Italy; 4 Institute for Genetic and Biomedical Research (IRGB), National Research Council (CNR), Milan, Italy; 5 Department of Precision Medicine, University of Campania Luigi Vanvitelli, Naples, Italy; 6 Department of Pediatrics, Stanford Medicine Children’s Health; Lucile Packard Children’s Hospital, Stanford, CA US; 7 Laboratory of Medical Genetics, Translational Cytogenomics Research Unit, Bambino Gesù Children Hospital-IRCCS, Rome, Italy; 8 Unit of Medical Genetics and Neurogenetics, Fondazione IRCCS Istituto Neurologico Carlo Besta, Milan, Italy; 9 Service de Génétique Médicale, CHU Nantes, Nantes, France; 10 Université de Nantes, CNRS, INSERM, L’Institut du Thorax, Nantes, France; 11 Department of Molecular Medicine and Medical Biotechnologies, University “Federico II”, Naples, Italy; 12 Department of Translational Medicine, University “Federico II”, Naples, Italy

**Keywords:** Genetics research, Clinical genetics

## Abstract

Mitochondrial disorders (MDs) are a diverse group of genetic conditions primarily affecting the oxidative phosphorylation (OXPHOS) system and cellular energy production. Among MDs, Linear Skin Defects with Multiple Congenital Anomalies (LSDMCA), or Microphthalmia with Linear Skin Lesions (MLS) syndrome, is a rare X-linked dominant male-lethal disorder characterized by ocular malformations, linear skin defects, and multisystem developmental anomalies. These features are associated with pathogenic variants in genes related to mitochondrial function, including *HCCS*, *COX7B*, and *NDUFB11* or chromosomal rearrangements of the Xp22 region encompassing *HCCS*. Despite progress, genotype-phenotype correlations remain insufficiently defined. In this study, we report three novel mutations in three patients with LSDMCA, broadening the phenotypic spectrum of the disorder. Whole exome sequencing revealed pathogenic missense variants in *HCCS* [NM_005333.5: c.625 G > C; p.(Asp209His)] and *COX7B* [NM_001866.3: c.221 C > T; p.(Pro74Leu)] in two unrelated patients. Functional studies confirmed that the COX7B variant impairs mitochondrial respiratory chain (MRC) function. A third patient harbored a novel frameshift pathogenic variant in *NDUFB11* [NM_001135998.3: c.145_152dup; p.(Thr52Glnfs*66)], further implicating mitochondrial dysfunction in LSDMCA pathogenesis. Notably, the *COX7B* variant was identified in a biological male (46, XY) without X-chromosome structural rearrangements, marking the first such reported case of LSDMCA. Our data suggest that certain missense variants, resulting in mild impairment of the gene product, may allow male survival, thereby expanding the known phenotype of this rare disorder. This report advances our understanding of genotype-phenotype correlations in LSDMCA and highlights the impact of mitochondrial dysfunction during embryonic development.

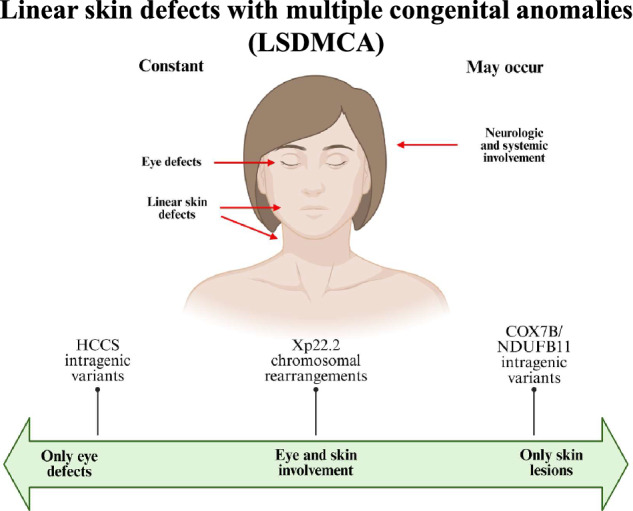

## Introduction

Mitochondrial disorders are clinically and genetically heterogeneous, with an estimated incidence of ~1:5000 [1]. These diseases can be caused by variants in either the nuclear or the mitochondrial genome, mainly resulting in impaired activity of the oxidative phosphorylation (OXPHOS) system [2]. Within mitochondria, OXPHOS converts the energy stored in nutrients (sugars, proteins, fats) into adenosine triphosphate (ATP), which then provides energy to power other cellular processes. This operation requires complex molecular machinery including five multimeric enzymes, known as MRC complexes I-V; coenzyme Q and cytochrome c shuttle the electrons from complex I or II to complex III, and from complex III to complex IV, respectively [3]. Holocytochrome C synthetase (HCCS) is a 268-amino-acid protein localized on the outer surface of the inner mitochondrial membrane. It catalyzes the covalent attachment of heme moieties to both cytochrome c and cytochrome c1, which are crucial components of the MRC: Cytc1 (an integral component of complex III) transfers the electrons to Cytc, which, in turn, transfers the electrons from complex III to complex IV [4, 5].Complex IV, also known as Cytochrome c oxidase (COX), evolved as the final oxygen accepting enzyme of the MRC [6]. In mammals, this complex is composed by three mitochondrial DNA-encoded subunits (COX1, COX2 and COX3) and ten nuclear genome-encoded ones (COX4, COX5A, COX5B, COX6A, COX6B, COX6C, COX7A, COX7B, COX7C and COX8) [7]. The *NDUFB11* gene encodes one of the 30 supernumerary subunits of NADH:ubiquinone oxidoreductase, the MRC complex I [8, 9]. Complex I represents the entry point for electrons in the MRC; this macromolecular complex oxidizes NADH to NAD^+^ in the mitochondrial matrix and supplies reducing equivalents to support the Krebs cycle and fatty acid β-oxidation [10].

Linear Skin defects with multiple congenital anomalies (LSDMCA), also known as microphthalmia with linear skin lesions (MLS) syndrome (MIM #309801; 300887; 300952) is a rare, X-linked dominant mitochondrial disorder [11, 12]. Although mitochondrial diseases are commonly associated with postnatal organ failure, LSDMCA is characterized by developmental anomalies of the eye, skin, and internal organs. In 1990, Temple and colleagues first described a female patient with microphthalmia, sclerocornea and linear aplastic lesions located on face and neck. An Xp22.2-Xpter deletion was found in the patient [13]. Since then, an additional 106 cases have been described [11, 12]. LSDMCA is almost invariably lethal in males [14]. To date, 11 cases have been reported of LSDMCA in patients who appeared phenotypically male, but all of them were found to be carriers of chromosomal rearrangements involving chromosomes Xp22.2 and chromosome Y [15–17].

LSDMCA typically presents with bilateral microphthalmia or even complete anophthalmia, which are evident at birth in most affected subjects, although a high degree of inter- and intrafamiliar variability of the phenotype exists [11]. The high degree of variability observed in this condition has been linked to chromosomal mosaicism and X chromosome inactivation schemes [12]. Common clinical features include sclerocornea, agenesis of corpus callosum, microcephaly, developmental delay, intellectual disability, and heart malformations/arrhythmias. A few cases displaying also diaphragmatic hernia and hearing impairment have been reported [12].

Molecularly, nearly 90% of reported cases involve chromosomal abnormalities at Xp22 [18]. The involved region contains, among others, the *HCCS* gene. A milestone in studying LSDMCA was the finding that overexpression of the human *HCCS* gene fully rescued embryonic lethality in mice with Xp22.2 deletions, strongly suggesting *HCCS* as the disease-causing gene [19]. Accordingly, pathogenic variants in *HCCS* have been found in several cases of LSDMCA [14, 20–24]. Cytochrome c is required for electron transport in mitochondria, thus functionality of *HCCS* is essential for proper activity of the MRC. Moreover, a critical role for HCCS in the control of apoptosis activation has been described. Mutations that reduce HCCS activity result in increased mitochondria-mediated cell death in the brain and eyes, further harming the affected tissues [5]. No other clinical phenotypes have been linked to variants involving *HCCS*.

Intragenic variants in the *COX7B* [25] and *NDUFB11* [8] genes, located as well on chromosome X, have been reported in few patients with LSDMCA. Like *HCCS*, *COX7B* and *NDUFB11* are genes involved in the MRC. Functional studies have shown that COX7B is necessary for COX activity and assembly, and MRC function [25]. In addition, a morpholino-based approach demonstrated in vivo an important function for complex IV activity in vertebrate brain and eye development [26]. Beside LSDMCA, pathogenic variants in *NDUFB11* have been also linked to Mitochondrial complex I deficiency, nuclear type 30 (MIM#301021) [27], histiocytoid cardiomyopathy [28] and early-onset sideroblastic anemia [29]. *NDUFB11* knockdown in HeLa cells caused impaired cell growth and increased apoptosis [8]. Interestingly, increased apoptosis has been also observed in in vivo models of *HCCS* and *COX7B* downregulation [5, 25].

Building on the recent advances in the understanding of the molecular basis of LSDMCA, the disorder has been further delineated in three different nosological entities: LSDMCA type 1, due to *HCCS* variants, LSDMCA type 2, due to *COX7B* variants, and LSDMCA type 3, due to variants involving *NDUFB11* [12].

To date, only a few genotype-phenotype correlations have been established for LSDMCA, and little is known about its pathogenic mechanisms.

By reporting two new cases and re-evaluating a previously published patient, in this work, we expand the clinical spectrum of LSDMCA and provide novel insights into how genetic alterations determine the phenotype of this rare disease.

## Materials and methods

### DNA sequencing, variant identification, and classification

Peripheral blood samples were taken from patients 1–3 and their parents to extract genomic DNA. For patient 1, custom clinical exome sequencing (Twist Bioscience, 6920 genes associated with genetic disorders) was performed on the Illumina NovaSeq6000. Reads were aligned to GRCh37/UCSC hg19; variants were called with the BaseSpace pipeline and annotated using Geneyx. Global minor allele frequencies were obtained from Genome Aggregation Database (gnomAD).

For patient 2, whole exome sequencing (Agilent SureSelect Clinical Research Exome XT, Illumina HiSeq 2500) generated 10.7 Gb of sequence with a mean 121× coverage of RefSeq coding bases, aligned to both nuclear and mitochondrial genomes.

For patient 3, Solo whole-exome sequencing was performed using the Twist X2 Human Core EF kit (Illumina NovaSeq6000, paired-end 2×150, ≥75× coverage). Reads were aligned to GRCh37/hg19 with BWA. Variants were called using GATK, CNVs with CANOES, and results were annotated using an in-house pipeline.

Segregation of the variants within the family was confirmed through Sanger sequencing.

For all patients, variants were evaluated by VarSome [30] and categorized in accordance with the American College of Medical Genetics and Genomics (ACMG) recommendations [31].

### Functional analysis of *COX7B* variant

Fibroblasts were obtained from patient skin biopsies and cultured under standard conditions in DMEM (EuroClone) with 10% FBS, split every 2–3 days. MRC complex I and IV activities were measured spectrophotometrically in digitonin-treated cells and normalized to citrate synthase (CS) activity; three technical replicates were analyzed and comparisons were performed using the Student’s t-test. For Western blot analysis, fibroblasts from patients and age- and sex-matched controls were lysed in RIPA buffer with protease inhibitors, quantified by Bradford assay, and probed with anti-COX7B (1:500, Abcam, ab137094) and anti-beta tubulin (1:1000, Cell Signaling) antibodies. Western blot band quantification was performed using Fiji ImageJ software.

### Data collection and analysis

Information for all patients previously described with LSDMCA was gathered from the literature through a comprehensive literature review. The PubMed database was searched up to August 1, 2025, using the following search terms and their combinations: “LSDMCA”, “microphthalmia with linear skin lesions”, “MLS syndrome”, “MIDAS syndrome”, “HCCS”, “COX7B”, and “NDUFB11”, “Xp22.2 deletion”. No restrictions on publication date were applied; only articles published in English were considered. Studies were included if they reported individual patients with a clinical diagnosis consistent with LSDMCA and provided either molecular confirmation (pathogenic variants in *HCCS*, *COX7B*, or *NDUFB11*) or documented chromosomal rearrangements involving Xp22.2 encompassing *HCCS*. Reports lacking sufficient clinical detail or without genetic or cytogenetic confirmation were excluded from genotype–phenotype analyses.

When multiple publications referred to the same patient, cases were identified based on overlapping clinical, genetic, and demographic features, and counted only once. The full list of patients and corresponding literature references is available in Supplemental Table 1. Data were analyzed using GraphPad Prism 5.0 software (San Diego, CA, USA). Comparisons of continuous variables between two and more experimental groups were performed using the two-tailed unpaired Student’s t-test or one-way ANOVA with Tukey’s post hoc tests, respectively. P-values < 0.05 were considered statistically significant.

## Results

### Patient 1

**Fig. 1:**
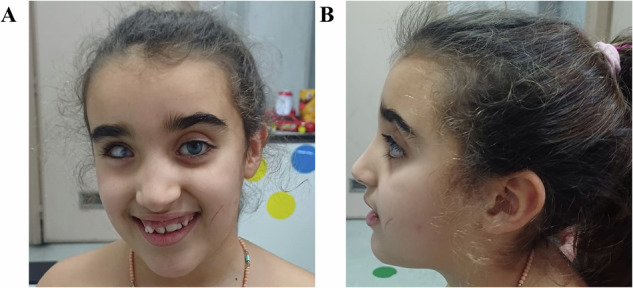
Physical examination of patient 1. **A** Frontal picture of the proband. Dental crowding is evident. **B** Lateral picture of the proband.

Patient 1 was a biological female born at full term following a spontaneous, uncomplicated pregnancy to healthy, non-consanguineous parents (birth weight 2,900 g; length 49 cm). At birth, bilateral corneal opacity was noted; further examination at Moorfields Eye Hospital (London, UK) confirmed bilateral, asymmetric sclerocornea, with cataract and glaucoma excluded. Psychomotor development was normal. At 23 months, clinical genetics evaluation revealed mild right microphthalmia and normal growth. At age 7, she exhibited bilateral corneal opacity, mild microphthalmia, high-arched palate, dental crowding (Fig. 1A, B), *genu valgum*, lumbar hyperkyphosis, ulnar deviation, valgus hindfoot, sandal gap, and tapering fingers; no skin lesions were observed, apart from mild *livedo reticularis*. Psychomotor development, school abilities and growth appeared normal.

Brain MRI scan showed flattened posterior profile of the eyeball, bilateral reduction in its convexity and a tortuous course of the optic nerves in their intra-orbital segment. All these alterations were more evident in the left eye. No contrast enhancement abnormalities were observed. Heart ultrasonography (US) highlighted patent foramen ovale (PFO) and mild left-right shunting. US of the abdominal organs did not show abnormalities. Transient-evoked otoacoustic emission test was normal.

**Fig. 2:**
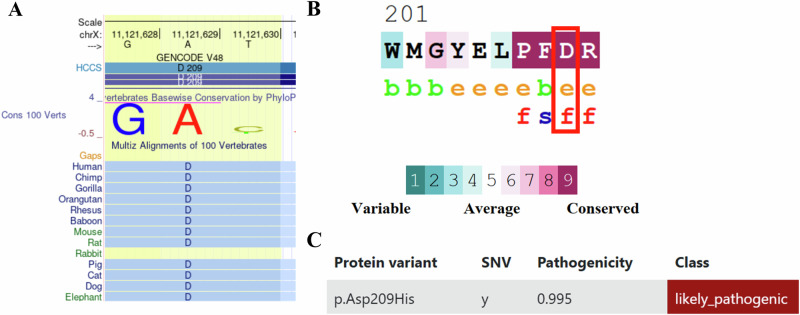
Asp209 is highly conserved in HCCS. **A** Multiple sequence alignment of *HCCS* gene with its vertebrate orthologs demonstrating that Aspartate at position 209 is strictly conserved across species. **B** ConSurf predicted conservation score for Aspartate 209 in HCCS, highlighted in the red box. Letter e indicates an amino acid predicted to be exposed on the surface of the protein, letter f indicates a predicted functional residue (highly conserved and exposed). **C** AlphaMissense predicted pathogenicity of p.Asp209His variant in *HCCS*.

SNP-array did not highlight chromosomal abnormalities worth further investigation. Trio clinical exome sequencing revealed a de novo missense variant in the *HCCS* gene [NM_005333.4: c.625 G > C; p.(Asp209His)]. The variant was not found in the gnomAD database and has never been described in ClinVar. The amino acid Asp209 is highly evolutionarily conserved (Fig. 2A), as confirmed by the ConSurf prediction tool [32], and it is predicted to play a highly functional role for the protein encoded by *HCCS* (Fig. 2B). Accordingly, AlphaMissense analysis indicates that variants at Asp209 are likely pathogenic (pathogenicity score: 0.995) [33] (Fig. 2C), and the p.Asp209His substitution is predicted to have a deleterious effect on protein function (PROVEAN score −6.942; CADD score: 27.1; REVEL score: 0.95; SIFT score: 0) [34–37]. For this reason, the variant was reported as likely pathogenic according to ACMG criteria (PS2, PM2, PP3) [31]. Accordingly, the patient received the diagnosis of LSDMCA type 1, consistent with the presence of unilateral microphthalmia, one of the two major criteria proposed for clinical diagnosis [11, 38].

### Patient 2

Patient 2 was a biological male born at term by planned cesarean section following a pregnancy complicated by well-controlled gestational diabetes. Birth weight was 3.1 kg and length 48 cm. Early development was delayed, with hypotonia and increased ankle reflexes noted at 16 months, and independent walking achieved at 24 months. At 25 months, mild dysmorphic features were observed (telecanthus, epicanthal folds, broad nasal bridge, anteverted nares, mildly tented upper lip, widely spaced teeth); no skin or eye abnormalities were present. He underwent unilateral orchidopexy at age 8. At 13 years, growth parameters were normal; he is currently non-verbal, uses gestures and an assistive device, and is partially independent in daily activities, with no history of seizures or behavioral issues.

A brain MRI performed at 23 months was normal. Follow up brain MRI scans at ages 3 years and 4 years showed a small cerebellum but no evidence for true atrophy on serial imaging.

High-resolution karyotype, array CGH, fragile X DNA, an X-linked Intellectual Disability NGS sequencing and mitochondrial DNA sequencing analysis were normal.

**Fig. 3:**
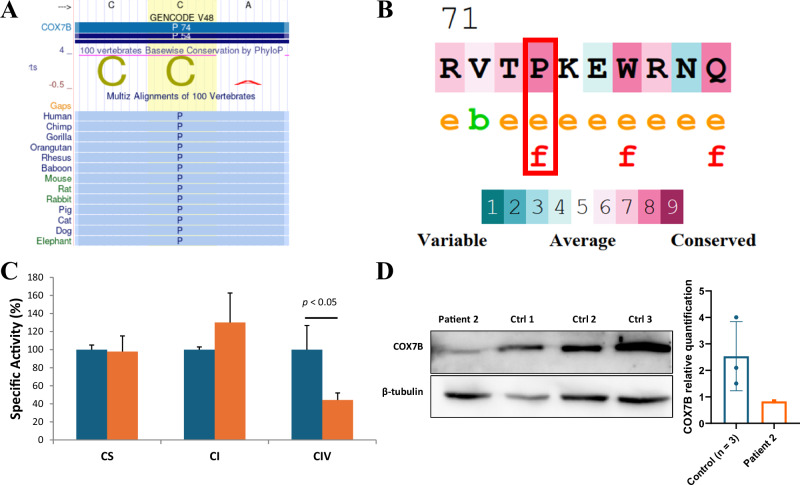
Functional impact of COX7B mutation. **A** Multiple sequence alignment of *COX7B* gene with its vertebrate orthologs demonstrating that Proline at position 74 is a strictly conserved amino acid across species. **B** ConSurf predicted conservation score for Proline 74 in COX7B, highlighted in the red box. Letter e indicates an amino acid predicted to be exposed on the surface of the protein, letter f indicates a predicted functional residue (highly conserved and exposed). **C** Mitochondrial Respiratory Chain Complexes specific activity; light gray bars indicate COX7B mutant, dark gray bars indicate controls. CS: citrate synthase, CI: Complex I, CIV: Complex IV. Three technical replicates were analyzed; Student’s T-test was used for comparisons; **D** Western blotting performed on whole cell lysates from patient- and healthy controls-derived fibroblasts. Beta tubulin was used as a loading control. COX7B relative quantification was performed using Fiji ImageJ.

Maternal X-inactivation studies showed random X-inactivation. Metabolic screening tests including lactate, ammonia, plasma amino acids, acylcarnitine profile, carnitine levels, urine organic acids and glycosylated transferrin, resulted normal. Finally, trio whole exome sequencing revealed a de novo variant [NM_001866.3:c.221 C > T; p.(Pro74Leu)] in the *COX7B* gene. The variant was not found neither in the gnomAD database nor in ClinVar and was predicted as likely pathogenic according to ACMG criteria (PS2, PM2). In silico analysis yielded conflicting results: AlphaMissense predicted the p.Pro74Leu variant as having an “ambiguous” effect on *COX7B*; similarly, the REVEL score of 0.45 fell into the range considered “uncertain”. In contrast, several other bioinformatic tools supported a potentially deleterious effect of the variant (CADD score: 23; PROVEAN score: −4.72; SIFT score: 0.04). Interestingly, recently developed variant effect predictor FuncVEP, whose predictions are entirely based on functional predictions, predicted the variant effect as strongly damaging (functional scores > 0.9) [39]. The amino acid Pro74 is highly conserved during evolution (Fig. 3A), and this was confirmed by the ConSurf prediction tool, which predicted to play a functional role (Fig. 3B).

### COX7B variant impairs complex IV activity

To test the effect of the c.221 C > T; p.(Pro74Leu) variant in *COX7B* gene, MRC activity was assayed in skin fibroblasts from patient 2 and healthy control. CS, an index of mitochondrial content in cells, was used for normalization.

While no alterations were observed in Complex I activity (28.9 mmol/min/mg; reference range: 15–32) Complex IV activity was reduced (60.27 mmol/min/mg; control range: 95–210), corresponding to an approximately 60% decrease relative to controls (Fig. 3C). These data indicate that the c.221 C > T;p.(Pro74Leu) variant identified impairs CIV activity. Notably, western blot analysis showed reduced protein abundance of COX7B in Patient 2 compared to healthy controls (Fig. 3D).

These findings suggest that the p.Pro74Leu variant has a negative impact on COX7B stability and may underlie the patient’s phenotype. Accordingly, variant prediction was changed to pathogenic according to ACMG criteria (PS2, PS3, PM2).

### Patient 3

Patient 3, a biological female, was previously reported [40, 41]. She presented with linear skin lesions on her face and neck, mild intellectual disability, microcephaly behavioral disturbance and facial dysmorphisms. Her growth was normal and she showed no eye abnormalities. Karyotype analysis resulted normal (46,XX). After previous clinical descriptions, her diagnosis was further pursued performing trio whole exome sequencing which revealed a de novo variant [NM_001135998.3:c.145_152dup (p.Thr52Glnfs*66)] in the *NDUFB11* gene. The variant was not found in the gnomAD database. The 7-nucleotide insertion was predicted to have a damaging effect on the protein and was reported as pathogenic according to ACMG criteria (PVS1, PS4, PM2, PP5).

## Discussion

LSDMCA is a rare genetic disease. At the time of writing, 109 cases, including those described in this paper, have been reported in the literature. Diagnosis is based on a combination of clinical features:

- micro/anophthalmia
- sclerocornea
- linear skin defects mostly located in the face or neck

Molecular genetics findings (chromosomal alterations or intragenic mutations involving at least one of the three genes *HCCS, COX7B, NDUFB11*) are used to confirm the diagnosis.

We have summarized clinical features of the disease in Table 1. The list of papers reviewed in this manuscript is shown in Supplemental Table 1. Almost all patients fulfill at least one of the two major criteria for the diagnosis of LSDMCA proposed by Happle [38]. Among the ocular features, we found that 80% of patients present with micro/anophthalmia and two thirds of them with sclerocornea/corneal opacity. Skin defects are observed in almost 85% of patients. The most involved area of the skin is the face, with reticulolinear lesions observed in 70% of patients. Lesions of the neck are present in one half of patients, while other body parts are less frequently involved (less than 20% of patients).

**Table 1:** Phenotypic features of LSDMCA.

Phenotype	Xp22.2 rearrangements	*HCCS* variant*s*	*COX7B* variants	*NDUFB11* variants
Eye defects	77/89	12/12	0/5; *p* = 0.0001	0/3; *p* = 0.0036
Micro/anophthalmia	76/89	12/12	0/5;*p* = 0.0002	0/3; *p* = 0.0045
Sclerocornea	58/89	11/12	0/5;*p* = 0.0069	0/3;*p* = 0.0477
Linear skin defects	78/89	4/12; *p* = 0.0002	4/5	3/3
ID/Developmental delay	19/89	2/12	3/5	2/3
Dysmorphisms	41/89	2/12	4/5	0/3
CNS involvement	44/89	4/12	1/5	2/3
Corpus callosum agenesis/hypoplasia	27/89	2/12	1/5	1/3
Heart involvement	39/89	6/12	2/5	2/3
Hearing loss	4/89	1/12	0/5	0/3
Anorectal malformations	12/89	0/12	0/5	0/3
Urogenital malformations	21/89	0/12	0/5	0/3
Diaphragmatic hernia	9/89	0/12	1/5	0/3

Patients carrying intragenic variants in *HCCS* have been compared with all patients diagnosed with the syndrome. Fisher’s exact test was performed to evaluate differences. P-value < 0.05 has been considered as statistically significant.

CNS involvement occurs in ~50% of patients, with intellectual disability and developmental delay seen in 10–20%; in our cohort, only one patient had mild intellectual disability, while Patients 1 and 2 developed normally. Agenesis of the corpus callosum occurs in ~30%, highlighting the role of mitochondrial OXPHOS in its development [42]. Notably, this finding has not been previously reported in the brain MRI imaging of mitochondrial diseases [43].

Dysmorphisms, short stature, and microcephaly affect 35, 18, and 12% of patients, respectively, including high-arched palate, low-set ears, anteverted nostrils, nail hypoplasia, facial asymmetry, and syndactyly.

Cardiac abnormalities occur in 40%, ranging from septal defects and patent foramen ovale/ductus arteriosus to arrhythmias, hypertrophic cardiomyopathy and complex malformations. Anorectal and urogenital malformations are reported in ~10% and 20% of patients, respectively, and diaphragmatic hernia, potentially life-threatening, in ~10% [14].

Only three patients have not presented with either micro/anophthalmia or linear dermal aplasia, one of whom is reported in this paper (patient 2) [5, 15]. Importantly, though, one of them showed sclerocornea and had a molecular genetics confirmation, suggesting that this finding should be combined with other eye abnormalities in the major criteria for establishing the diagnosis of LSDMCA.

We divided all patients reported in literature based on the genetic cause for their disease into four groups: patients with intragenic mutations in *COX7B* [25], patients with intragenic mutations in *NDUFB11* [8, 40], patients with intragenic mutations in *HCCS* and patients with Xp22.2 chromosomal rearrangements involving *HCCS*.

**Fig. 4:**
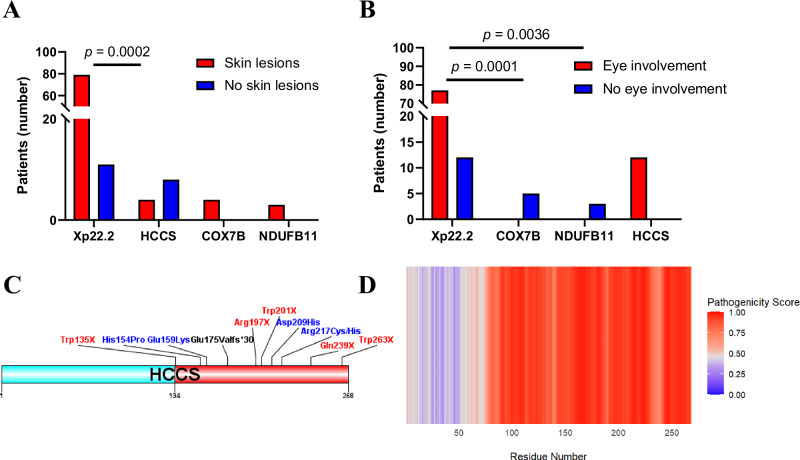
Clinical and genetic features of LSDMCA. **A** Prevalence of skin lesions in all subgroups of patients with diagnosed LSDMCA; **B** prevalence of eye involvement in all subgroups of patients with diagnosed LSDMCA. Xp22.2, patients with chromosomal rearrangements involving chromosome Xp22.2; HCCS, patients with intragenic variants in *HCCS*; COX7B; patients with intragenic variants in *COX7B*; NDUFB11, patients with intragenic variants in *NDUFB11*. Fisher’s exact test was used for statistical analysis; **C** Graphic depiction of all known intragenic variants in *HCCS* found in LSDMCA patients. Highlighted in dark gray, the amino acids 135-268 that represent the C-terminal half of the protein, where all known intragenic mutations in *HCCS* have been found. Written in light gray, nonsense mutations; in dark gray, missense mutations; in black, frameshift mutations. The image has been produced using DOG2.0 software [49]; **D** Pathogenicity confidence heatmap showing the predicted pathogenicity of amino acidic substitutions involving any of the 268 amino acids of HCCS, as predicted by AlphaMissense; in blue, likely benign variants; in gray, variants of uncertain significance; in red, likely pathogenic variants.

We observed that, although reported in very few patients, variants in *COX7B* and *NDUFB11*(patients with LSDMCA type 2 and 3) are associated to skin defects (7/8 patients) with no or very limited involvement of the eye (0/8 patients). This finding is in contrast with the overall prevalence in all known patients with LSDMCA being 89/109 (*p* = 0.0001 for *COX7B* mutations, *p* = 0.0036 for *NDUFSB11* mutations, Fisher’s exact test) (Fig. 4A). Intriguingly, the only patient who did not show skin lesions was our Patient 2, who was the only patient with a missense, rather than nonsense, variant.

By contrast, patients with intragenic variants in *HCCS* (LSDMCA type 1) invariably show eye defects (12/12 patients) but only 4/12 have skin involvement, the overall prevalence of patients with skin lesions being 89/109, meaning that LSDMCA without skin involvement is more than 4 times more common in patients who bear intragenic variants in *HCCS*, with a statistically significant enrichment (*p* = 0.0007, Fisher’s exact test) (Fig. 4B). These data confirm a previous report based on a smaller sample size [12]. No other specific features were observed in patients with LSDMCA type 1. In two patients, the agenesis of septum pellucidum was described, together with hypoplasia of corpus callosum [14, 23]. Notably, both patients carried the same variant in *HCCS* c.589 C > T (p.Arg197*). No other significant differences among the subgroups of patients arose from our analysis.

To date, it is still unclear why skin involvement is significantly less frequent in patients with LSDMCA type 1. It has been proposed that the reduced prevalence of skin involvement in patients with ocular anomalies carrying *HCCS* variants might be due to ascertainment bias [22]. However, cutaneous lesions and scars in patients with LSDMCA are typically quite apparent upon routine clinical inspection, a feature further facilitated by their common localization on the face and the neck of patients. While it is theoretically possible that patients presenting to an ophthalmology center for ocular defects may sometimes be overlooked for manifestations non directly involving the eyes, it is important to note that we did not find any difference in the prevalence of internal anomalies—including CNS and heart malformations, which may be asymptomatic in several cases—between patients with intragenic *HCCS* variants and the broader cohort of patients diagnosed with LSDMCA (see Table 1). It should be noted that chromosome Xp22.2 deletions, which are frequently associated with skin lesions (78/89 patients), encompass the *HCCS* gene. Consequently, the apparently reduced prevalence of skin lesions in individuals with intragenic *HCCS* variants may reflect an artifact rather than a true phenotypic difference. Clarification of this observation will likely emerge as additional cases of LSDMCA are reported.

All known *HCCS* intragenic mutations found in LSDMCA type 1 patients involve amino acids predicted to be located into secondary structures (alpha-helices, beta sheets) or in collecting loops between them; all identified mutations are confined to the C-terminal half of the protein. Remarkably, nearly all amino acid substitutions occurring within this region are predicted to be pathogenic based on assessments by AlphaMissense, highlighting the critical functional importance of the C-terminal domain (Fig. 4C, D). In particular, all missense mutations involve amino acids whose substitution is predicted to be highly pathogenic according to AlphaMissense (Fig. 4C, D). We hypothesize that intragenic variants in *HCCS* might disrupt the stability of the holocytochrome c synthetase enzyme and impair its catalytic activity. Another potential pathogenic mechanism may involve disruption of the interaction between holocytochrome c synthetase and the mitochondrial inner membrane. *HCCS* has been shown to be membrane-associated, although the determinants of this interaction are still unclear and no canonical transmembrane helices have been predicted in its structure [4].

LSDMCA is considered to be a male-lethal disorder; all patients reported so far had a female karyotype, although few of them presented with male genitalia [15–17], the only exception being one patient that was found to carry a mosaic paracentric inversion of chromosome Xp [44]. Importantly, this patient developed a severe phenotype although the chromosomal aberrations was detected in only 15% of his cells. Our report presents the first ever description of LSDMCA in a male patient with 46,XY karyotype and no chromosomal aberrations (Patient 2).

All patients described so far with *COX7B*-related LSDMCA had nonsense or frameshift variants in *COX7B* [25, 45], while Patient 2 had a missense variant (p.Pro74Leu). In vitro analysis showed reduced, but not abolished protein expression and CIV activity. We hypothesize that missense variants in *COX7B* determine a milder phenotype than nonsense and frameshift ones, thus “escaping” male lethality of LSDMCA. *DDX3X*-related neurodevelopmental disorder (DDX3X-NDD) is an X-linked disease characterized by developmental delay and intellectual disability mostly affecting females [46]. Male patients with DDX3X-NDD invariably present with missense variants, which produce very mild or no phenotype at all when detected in females. It has been proposed that nonsense variants observed in female patients may be incompatible with life if identified in males [47]. We hypothesize that a similar mechanism could occur in LSDMCA with *COX7B* variants, but further studies will be necessary to clearly understand the mechanisms of male lethality in LSDMCA. X chromosome inactivation (XCI) likely plays a role in modulating the clinical variability of LSDMCA; XCI usually occurs randomly, but in certain conditions a non-random XCI (skewed XCI) may occur, with preferential inactivation of the mutant X chromosome that may lead to milder clinical features of the disease. The extent of skewed XCI is likely responsible for the phenotypic variability observed in females with male-lethal disorders as LSDMCA [48].

LSDMCA can be viewed as a *continuum*, rather than a single disease, with relevant heterogeneity spanning from mild forms involving the eye and/or the skin without affecting any other organ to severe and male-lethal forms. Further analysis could contribute to elucidating the role of other genes as disease modifiers.

Mitochondrial disorders may frequently present with metabolic abnormalities [1]. The absence of detectable metabolic alterations in LSDMCA, despite impaired OXPHOS activity, may be attributable to the lack of analyses performed in disease-relevant tissues.

In conclusion, we have reported three new cases of LSDMCA types 1–3 with novel variants in *HCCS*, *COX7B* and *NDUFB11*. The first case shows isolated sclerocornea and mild cardiac malformations, without skin, CNS, psychomotor or intellectual involvement. Genetic testing uncovered a heterozygous previously unreported missense variant in *HCCS* c.625 G > C resulting in an amino acid substitution of Aspartate 209 with a Histidine, which is predicted to be likely pathogenic. We have then reported a male patient with LSDMCA type 2 only showing mild intellectual disability and psychomotor delay, facial dysmorphisms and cryptorchidism, without any eye or skin involvement. This patient was found to carry a missense variant, initially classified as likely pathogenic, in the *COX7B* gene. Functional analysis provided decisive evidence that the variant is disease-causing. A third patient, who had been previously described from a clinical point of view [40, 41] with no genetic diagnosis was finally found to bear a pathogenic frameshift variant in the *NDUFB11* gene and diagnosed with LSDMCA type 3.

By critically reviewing the available evidence in literature, we found that intragenic variants in *HCCS* are often linked to LSDMCA without skin lesions; we also found that neurodevelopmental delay and intellectual disability are infrequent in LSDMCA type 1, with only one patient showing mild intellectual disability and one presenting with mild motor delay [14, 23]. By contrast, we found that ocular involvement does not seem to occur in LSDMCAs types 2 and 3, which almost invariably present with skin lesions; interestingly, the only known case to date with a missense variant in *COX7B* also represents the only patient with no skin lesions. *COX7B* and *NDUFB11* variants may be more frequently associated with neurodevelopmental delay; however, the small sample size limits statistical conclusions. We reported the first male (46,XY, without chromosomal abnormalities) patient ever diagnosed with LSDMCA. We hypothesize that male lethality may be avoided due to the mild reduction in enzyme activity caused by the missense variant in *COX7B*. These findings might help clarify the mechanisms of male lethality in LSDMCA and related disorders.

Our observations might represent a first step towards a more precise stratification of LSDMCA patients based on genotype-phenotype correlations.

## Supplementary information

Supplemental Table 1

## Data Availability

This study used individual data from published papers available in Pubmed and reported in Supplemental table 1. Individual data regarding the patients clinically evaluated by the authors are available upon request.
