# Supplementary material for: Revisiting LSDMCA: male lethality escape and genotype-phenotype correlations
Source: Eur J Hum Genet. 2026 Apr 21;34(7):972–9. doi: 10.1038/s41431-026-02098-7 (PMC13342626; doi:10.1038/s41431-026-02098-7)
Supplement: Supplementary file 1 — Supplemental Table 1 [file 41431_2026_2098_MOESM1_ESM.docx]

| **First author** | **Year** | **PMID/doi** | **Patients** | **Sex** |
| --- | --- | --- | --- | --- |
| Temple | 1990 | 2308156 | 1 | F |
| Allanson | 1991 | 2002490 | 2 | F |
| Lindor | 1992 | 1519653 | 1 | F |
| Naritomi | 1992 | 1642272 | 2 | F |
| Al-Gazali | 1993 | 2308157 | 2 | F |
| Happle | 1993 | 8267001 | 1 | F |
| Bird | 1994 | 7856638 | 1 | F |
| Eng | 1994 | 8089303 | 1 | F |
| Lindsay | 1994 | 8116674 | 3 | 2F, 1 46XX M |
| Mucke | 1995 | 7645589 | 2 | F |
| Paulger | 1997 | 9050760 | 1 | 46 XX M |
| Cox | 1998 | 9781050 | 1 | F |
| Ogata | 1998 | 9737776 | 1 | F |
| Stratton | 1998 | 9508062 | 1 | 46XX M |
| Zvulunov | 1998 | 9747372 | 1 | F |
| Kono | 1999 | 9929982 | 1 | 46XX M |
| Kayserili | 2001 | 11424926 | 1 | F |
| Kutsche | 2002 | 12900578 | 1 | M |
| Paul | 2002 | 12108507 | 1 | F |
| Anguiano | 2003 | 12707958 | 2 | 2 46XX M |
| Enright | 2003 | 12657015 | 1 | F |
| Kherbaoui-Redouani | 2003 | 12829336 | 1 | F |
| Cape | 2004 | 15249380 | 4 | 2 46 XX M, 2F |
| Morleo | 2005 | 16059943 | 7 | 7 F |
| Wimplinger | 2006 | 17033964 | 5 | F |
| Cain | 2007 | 17286317 | 1 | F fetus |
| Schluth | 2007 | 16690229 | 1 | F |
| Wimplinger | 2007 | 17845869 | 1 | F |
| Wimplinger | 2007 | 17893649 | 1 | F |
| Kapur | 2008 | 18580270 | 2 | 1 F, 1 46XX M |
| Sharma | 2008 | 18950397 | 5 | F |
| Harmsen | 2009 | 19277062 | 3 | F |
| Hobson | 2009 | 19610109 | 1 | F |
| Steichen-Gersdorf | 2010 | 20179582 | 1 | F |
| Alberry | 2011 | 21200317 | 1 | F fetus |
| Indrieri | 2012 | 23122588 | 3 | F |
| Zumwalt | 2012 | 22409474 | 1 | F |
| Garcia-Rabasco | 2013 | 22612277 | 1 | F |
| Vergult | 2013 | 23401659 | 2 | F |
| Almeida | 2014 | 24626674 | 1 | F |
| Herwig | 2014 | 25291437 | 1 | F fetus |
| Kluger | 2014 | 24096629 | 1 | F |
| Margari | 2014 | 25182979 | 1 | F |
| van Rahden | 2014 | 24735900 | 6 | F |
| van Rahden | 2015 | 25772934 | 2 | F |
| Weh | 2015 | 25182519 | 1 | F |
| Durack | 2018 | 29896851 | 1 | F |
| Kumar | 2018 | 29023962 | 2 | F |
| Prepeluh | 2018 | 30068298 | 1 | F |
| Thompson | 2018 | 29088057 | 1 | 47XXX F |
| Banganho | 2019 | 31015240 | 1 | F |
| Chateau | 2020 | 32386085 | 2 | F |
| Satcher | 2020 | 31373408 | 1 | F |
| Vendramini-Pittoli | 2020 | 32765930 | 1 | F |
| Piekutowska-Abramczuk | 2022 | 35893073 | 1 | F |
| Franco | 2023 | 36369709 | 5 | F |
| Mendiratta | 2023 | 10.4103/ijpd.ijpd_31_23 | 1 | F |
| Reis | 2024 | 39766903 | 7 | F |
| Singh | 2024 | 38038053 | 1 | 46XX M |
| Present study |  |  | 2 | 1F, 1M |
| **Total** |  |  | 109 |  |

**Supplemental Table 1:** List of patients and papers reviewed in this work.
